# An Alignment-Free Algorithm in Comparing the Similarity of Protein Sequences Based on Pseudo-Markov Transition Probabilities among Amino Acids

**DOI:** 10.1371/journal.pone.0167430

**Published:** 2016-12-05

**Authors:** Yushuang Li, Tian Song, Jiasheng Yang, Yi Zhang, Jialiang Yang

**Affiliations:** 1 School of Science, Yanshan University, Qinhuangdao, China; 2 Department of Civil and Environmental Engineering, National Universality of Singapore, Singapore; 3 Department of Mathematics, Hebei University of Science and Technology, Shijiazhuang, Hebei, China; 4 School of Mathematics and Information Science, Henan Polytechnic University, Henan, China; Tianjin University, CHINA

## Abstract

In this paper, we have proposed a novel alignment-free method for comparing the similarity of protein sequences. We first encode a protein sequence into a 440 dimensional feature vector consisting of a 400 dimensional Pseudo-Markov transition probability vector among the 20 amino acids, a 20 dimensional content ratio vector, and a 20 dimensional position ratio vector of the amino acids in the sequence. By evaluating the Euclidean distances among the representing vectors, we compare the similarity of protein sequences. We then apply this method into the ND5 dataset consisting of the ND5 protein sequences of 9 species, and the F10 and G11 datasets representing two of the xylanases containing glycoside hydrolase families, i.e., families 10 and 11. As a result, our method achieves a correlation coefficient of 0.962 with the canonical protein sequence aligner ClustalW in the ND5 dataset, much higher than those of other 5 popular alignment-free methods. In addition, we successfully separate the xylanases sequences in the F10 family and the G11 family and illustrate that the F10 family is more heat stable than the G11 family, consistent with a few previous studies. Moreover, we prove mathematically an identity equation involving the Pseudo-Markov transition probability vector and the amino acids content ratio vector.

## Introduction

With the recent development of next-generation sequencing technologies, there has been an explosion in the numbers of available DNA and protein sequences. The numerous newly sequenced protein sequences present an urgent need for novel computational algorithms to compare their similarities with sequences from known protein families, to predict their structures, and thus to infer their functions [1–6].

As usually the first step in a bioinformatics pipeline, sequence comparison is very crucial since it affects all down-stream analyses. Popular methods for sequence comparison generally fall into two categories: those using sequence alignment and those using alignment-free methods. In a sequence alignment, a score function is used to represent insertion, deletion, and substitution of nucleotides or amino acids in the compared DNAs or proteins, and the objective is to identity the alignment with the highest overall alignment score through methods like dynamic programming and seeding [7–9]. However, sometimes alignment becomes misleading due to unequal lengths of sequences, gene rearrangements, inversion, transposition, and translocation at substring level [10]. In these scenarios, alignment-free methods present good alternatives to alignment methods, which usually quantify sequence similarities using K-mer frequencies and other sequence features [11].

An alignment-free method for comparing protein sequences usually consists of two steps. At first, the protein sequences are transformed into fixed-length feature vectors [12]. The feature vectors are then fed into a vector similarity comparison algorithm to perform downstream analysis like phylogenetic inference [13]. Feature extraction is a procedure to extract desired information from the query sequences, which is usually critical to the accuracy of an alignment-free method [14]. Widely accepted features include chemical and physical properties [15], distance frequency matrix [16], K-string dictionary [17], 2D and 3D amino acid adjacency matrices [18], pseudo amino acid composition [19], and sequential and structural evolution information [20, 21]. Though these methods have their own advantages, they are suffering problems like computational intensive and low accuracy. Thus, more discriminatory features are still in demanding.

To further improve protein sequence comparison accuracy, we present a novel 440 dimensional feature vector, which models a few important information of a protein including the amino acids’ abundance and position information, and the Pseudo-Markov transition probabilities among them. We then test the performance of our feature vector in two well studied datasets: (1) the ND5 dataset [22] and (2) the F10 and G11 dataset [23]. They have been widely used in evaluating protein comparison algorithms [22, 24]. As a result, our method is more accurate than a few existing methods for similarity analysis on the ND5 dataset, and we achieve accurate phylogenetic tree and heat stability results on the F10 and G11 dataset.

## Method

Amino acid composition and distribution are two most fundamental information about a protein sequence. They have been widely used and proven to be effective in protein sequence analyses [25], structural classification [26–28], pattern recognition receptor prediction [29], and fold recognition [30]. Thus, we proposed a novel representation for a protein sequence based on the two features, i.e. a 440-D feature vector consisting of (1) a 400-D Pseudo-Markov transition probability vector reflecting the order information of adjacent amino acids. (2) a 20-D amino acid content ratio vector describing the frequency of each amino acid in the sequence, and (3) a 20-D amino acid position ratio vector exhibiting the position distribution of each amino acid.

### Construction of the 400 dimensional Pseudo-Markov transition probability vector

Let *S* = *S*_1_*S*_2_⋯*S*_*N*_ be a protein sequence of length *N* defined on *A* = {*A*_1_, *A*_2_, ⋯, *A*_20_}, an ordered alphabet of 20 amino acids. For 1 ≤ *i*, *j* ≤ 20, 1 ≤ *k* ≤ *N* and 1 ≤ *l* ≤ *N* − 1, an amino acid *A*_*i*_ is said to occur at position *k* if *S*_*k*_ = *A*_*i*_, and an ordered amino acid pair *A*_*i*_*A*_*j*_ is said to occur at position *l* if *S*_*l*_*S*_*l*+1_ = *A*_*i*_*A*_*j*_. Let *n*_*i*_ be the number of occurrences of *A*_*i*_ and *n*_*i*,*j*_ be the number of occurrences of *A*_*i*_*A*_*j*_ in *S*. We then define the 400 dimensional vector as (*P*_1,1_, *P*_1,2_, ⋯, *P*_1,20_, *P*_2,1_, *P*_2,2_, ⋯, *P*_2,20_, ⋯, *P*_20,1_, *P*_20,2_, ⋯, *P*_20,20_), where

$$P_{i,j}=\{\begin{array}{cc} \frac{n_{i,j}}{n_{i}} & \text{if }A_{i}\neq S_{N}\\ \frac{n_{i,j}}{n_{i}-1} & \text{if }A_{i}=S_{N} \end{array}$$ (1)

In particular, if there is no *A*_*i*_ or *A*_*i*_ appears only once at the end of *S*, then the numerator and denominator of *P*_*i*,*j*_ are both 0. In this case, we define *P*_*i*,*j*_ = 0.

By definition, we have

$$\sum_{j=1}^{20}n_{i,j}=\{\begin{array}{cc} n_{i} & \text{if }A_{i}\neq S_{N}\\ n_{i}-1 & \text{if }A_{i}=S_{N} \end{array}$$ (2)

$$\sum_{i=1}^{20}n_{i,j}=\{\begin{array}{cc} n_{j} & \text{if }A_{j}\neq S_{1}\\ n_{j}-1 & \text{if }A_{j}=S_{1} \end{array}$$ (3)

From eqs (1) and (2) we have $\sum_{j=1}^{20}P_{i,j}=1,$ and thus *P*_*i*,*j*_ can be considered as a transition probability from amino acid *A*_*i*_ to *A*_*j*_ in the protein sequence. So we call the 400 dimensional vector (*P*_1,1_, …, *P*_1,20_, *P*_2,1_, …, *P*_2,20_, …, *P*_20,1_, …, *P*_20,20_) a Pseudo-Markov transition probability vector.

### Construction of the 20 dimensional amino acid content ratio vector

Given that the protein sequence is composed of only 20 amino acids, it is clear that $\sum_{i=1}^{20}n_{i}=N$. For each amino acid *A*_*i*_ (1≤*i*≤20), we define its content ratio *C*_*i*_ as $C_{i}=\frac{n_{i}}{N}$ and the 20 dimensional amino acid content ratio vector as (*C*_1_, *C*_2_, …, *C*_20_). Obviously, $\sum_{i=1}^{20}C_{i}=1$.

### Construction of the 20 dimensional amino acid position ratio vector

For each amino acid *A*_*i*_ (1≤*i*≤20), let *s*_*i*_ be the sum of all positions in *S* that *A*_*i*_ occurs. Noticing that $\sum_{i=1}^{20}s_{i}=\frac{N(N+1)}{2}$, we define the position ratio of the amino acid *D*_*i*_ as $D_{i}=\frac{2s_{i}}{N(N+1)}$ and the 20 dimensional amino acid position ratio vector as (*D*_1_, *D*_2_, …, *D*_20_). Obviously, $\sum_{i=1}^{20}D_{i}=1$.

By concatenating the above three types of vectors, we obtain a 440-D feature vector of *S*, that is, *V*_*s*_ = (*P*_1,1_, …, *P*_20,20_, *C*_1_, …, *C*_20_, *D*_1_, …, *D*_20_). In the following, we show an interesting property of *V*_*s*_. For 1≤*j*≤20, let $\Delta_{j}=\sum_{i=1}^{20}C_{i}P_{i,j}$.

#### Property

Suppose *S*_1_ = *A*_*u*_ and *S*_*N*_ = *A*_*v*_ for indices *u* and *v* with 1≤*u*, *v*≤20. Then for any 1≤ *j* ≤20, we have

$$\Delta_{j}=\{\begin{array}{cc} C_{j}-\frac{1}{N}+\frac{P_{v,j}}{N} & \text{if }j=u\\ C_{j}+\frac{P_{v,j}}{N} & \text{if }j\neq u \end{array}$$

In particular, if *A*_*v*_ occurs only once in *S*, i.e. *n*_*v*_ = 1 then

$$\Delta_{j}=\{\begin{array}{cc} C_{j}-\frac{1}{N} & \text{if }j=u\\ C_{j} & \text{if }j\neq u \end{array}$$

#### Proof

If *j* = *u*, from eqs (1) and (3) we have

$$\begin{array}{l} \Delta_{u}=\sum_{i=1}^{20}C_{i}P_{i,u}=C_{1}P_{1,u}+C_{2}P_{2,u}+\cdots +C_{u}P_{u,u}+\cdots +C_{v}P_{v,u}+\cdots +C_{20}P_{20,u}\\ =\frac{n_{1}}{N}\times \frac{n_{1,u}}{n_{1}}+\frac{n_{2}}{N}\times \frac{n_{2,u}}{n_{2}}+\cdots +\frac{n_{u}}{N}\times \frac{n_{u,u}}{n_{u}}+\cdots +\frac{n_{v}}{N}\times \frac{n_{v,u}}{n_{v}-1}+\cdots +\frac{n_{20}}{N}\times \frac{n_{20,u}}{n_{20}}\\ =\frac{n_{1,u}}{N}+\frac{n_{2,u}}{N}+\cdots +\frac{n_{u,u}}{N}+\cdots +\frac{n_{v}}{N}\times \frac{n_{v,u}}{n_{v}-1}+\cdots +\frac{n_{20,u}}{N}\\ =\frac{n_{1,u}+n_{2,u}+\cdots +n_{u,u}+\cdots +n_{v,u}+\cdots +n_{20,u}-n_{v,u}}{N}+\frac{n_{v}}{N}\times \frac{n_{v,u}}{n_{v}-1}\\ =\frac{n_{u}-1-n_{v,u}}{N}+\frac{n_{v}}{N}\times \frac{n_{v,u}}{n_{v}-1}\\ =\frac{n_{u}}{N}-\frac{1}{N}+\frac{n_{v,u}}{N}(\frac{n_{v}}{n_{v}-1}-1)\\ =C_{u}-\frac{1}{N}+\frac{P_{v,u}}{N} \end{array}$$

If *j* ≠ *u*, we have

$$\begin{array}{l} \Delta_{j}=\sum_{i=1}^{20}C_{i}P_{i,j}=C_{1}P_{1,j}+C_{2}P_{2,j}+\cdots +C_{u}P_{u,j}+\cdots +C_{v}P_{v,j}+\cdots +C_{20}P_{20,j}\\ =\frac{n_{1}}{N}\times \frac{n_{1,j}}{n_{1}}+\frac{n_{2}}{N}\times \frac{n_{2,j}}{n_{2}}+\cdots +\frac{n_{u}}{N}\times \frac{n_{u,j}}{n_{u}}+\cdots +\frac{n_{v}}{N}\times \frac{n_{v,j}}{n_{v}-1}+\cdots +\frac{n_{20}}{N}\times \frac{n_{20,j}}{n_{20}}\\ =\frac{n_{1,j}}{N}+\frac{n_{2,j}}{N}+\cdots +\frac{n_{u,j}}{N}+\cdots +\frac{n_{v}}{N}\times \frac{n_{v,j}}{n_{v}-1}+\cdots +\frac{n_{20,j}}{N}\\ =\frac{n_{1,j}+n_{2,j}+\cdots +n_{u,j}+\cdots +n_{v,j}+\cdots +n_{20,j}-n_{v,j}}{N}+\frac{n_{v}}{N}\times \frac{n_{v,j}}{n_{v}-1}\\ =\frac{n_{j}-n_{v,j}}{N}+\frac{n_{v}}{N}\times \frac{n_{v,j}}{n_{v}-1}\\ =\frac{n_{j}}{N}+\frac{n_{v,j}}{N}(\frac{n_{v}}{n_{v}-1}-1)\\ =C_{j}+\frac{P_{v,j}}{N} \end{array}$$

Finally, let *n*_*v*_ = 1. By definition, we have *n*_*v*,*j*_ = 0 and *P*_*v*,*j*_ = 0 for any 1≤ *j* ≤20. Thus,

$$\Delta_{j}=\{\begin{array}{cc} C_{j}-\frac{1}{N} & \text{if }j=u\\ C_{j} & \text{if }j\neq u \end{array},$$

completing the proof.

### Quantifying the distances among protein sequences based on their feature vectors

Let *S* and *T* be two proteins and *V*_*S*_ and *V*_*T*_ be their 440-D feature vectors. Then the distance between *S* and *T* is quantified by the Euclidean distance between *V*_*S*_ and *V*_*T*_, that is, $d(S,T)=\sqrt{\sum_{i=1}^{440}{\left(V_{S}[i]-V_{T}[i]\right)}^{2}}$, where *V*_*S*_[*i*] and *V*_*T*_[*i*] denote the i^th^ entries of the vectors *V*_*S*_ and *V*_*T*_ respectively.

## Results and Discussions

To evaluate the performance of our method, we applied it into two datasets: (1) the ND5 dataset [22] and (2) the F10 and G11 dataset [23].

### Datasets

The ND5 dataset consists of the ND5 protein sequences of 9 species including human, gorilla, pigmy chimpanzee, common chimpanzee, fin whale, blue whale, rat, mouse, and opossum (Table 1). The sequences have lengths 602~610 base pairs (bps). It is a popular benchmark data for testing the performances of computational methods in comparing the similarity of protein sequences [15, 31–34].

**Table 1:** Information of ND5 for nine species.

Species	ID (NCBI)	Length
Human (Homo sapiens)	AP_000649	603
Gorilla (Gorilla gorilla)	NP_008222	603
Pigmy chimpanzee (Pan paniscus)	NP_008209	603
Common chimpanzee (Pan troglodytes)	NP_008196	603
Fin whale (Balenoptera physalus)	NP_006899	606
Blue whale (Balenoptera musculus)	NP_007066	606
Rat (Tattus norvegicus)	NP_004902	610
Mouse (Mus musculus)	NP_904338	607
Opossum (Didelphis virginiana)	NP_007105	602

The F10 and G11 datasets represent two of the xylanases containing glycoside hydrolase families, i.e., families 10 and 11 respectively. Specifically, the F10 dataset contains ten xylanases with NCBI accession IDs O59859, P56588, P33559, Q00177, P07986, P07528, P40943, P23556, P45703, and Q60041 respectively. The G11 dataset also consists of ten xylanases with NCBI IDs P33557, P55328, P55331, P45705, P26220, P55334, Q06562, P55332, P55333, and P17137 respectively.

### Application to the ND5 dataset

**Fig 1:**
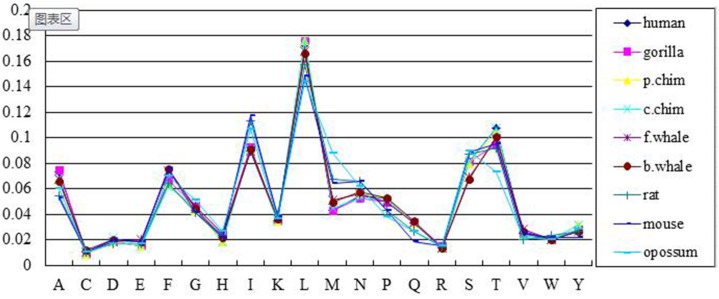
The content ratios of twenty amino acids in the ND5 dataset. The X axis denotes the 20 amino acids and the Y axis denotes the content ratios of each amino acid for the 9 sequences.

**Fig 2:**
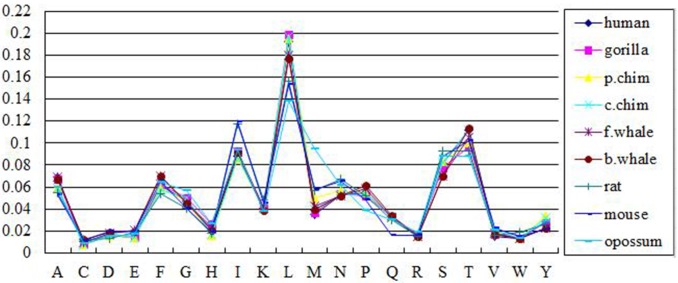
The position ratios of twenty amino acids in the ND5 dataset. The X axis denotes the 20 amino acids and the Y axis denotes the position ratios of each amino acid for 9 sequences.

We first encoded the nine protein sequences into 440-D feature vectors. In Figs 1 and 2, we showed the content ratios and position ratios of the twenty amino acids over the sequences.

As can be seen, the content ratios and position ratios exhibit similar trends over the twenty amino acids. The amino acid L has the highest content ratio and position ratio over all 9 sequences whereas amino acid C has the lowest content ratio and position ratio. In addition, the 9 species are quite similar according to the amino acids distributions of both the content ratio and position ratio in the ND5 protein.

**Fig 3:**
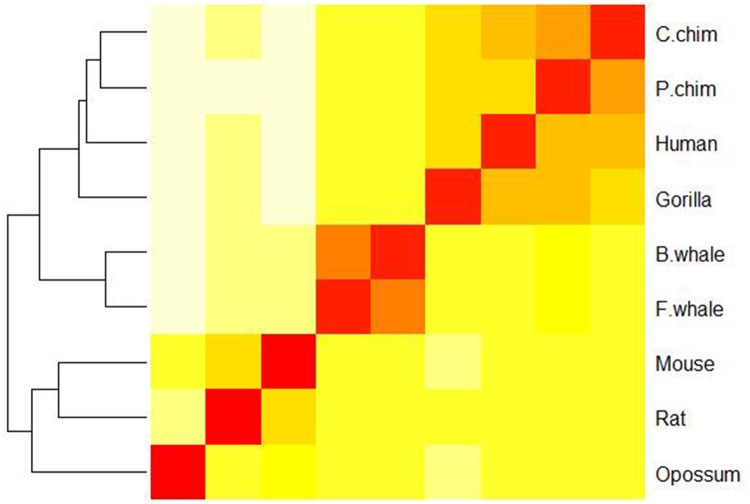
A heat map showing the similarity of nine species in the ND5 dataset. Red color indicates small distance and high similarity between the sequences and yellow color indicates large distance and low similarity, the same as below.

We then calculated the pairwise Euclidean distances among the nine 440-D feature vectors and showed the results in Table 2. As we can see, human, P.chim, C.chim, and gorilla are closer to each other and they are relatively far from rat, mouse and opossum. For a better view, we also plotted a heat-map based on the distances in Fig 3.

**Table 2:** The distance matrix of nine species by our method.

	Human	Gorilla	P.chim	C.chim	F.whale	B.whale	Rat	Mouse	Opossum
**Human**	0	0.53	0.497	0.501	0.764	0.782	0.901	0.945	0.972
**Gorilla**		0	0.522	0.564	0.755	0.803	0.876	0.963	1.025
**P.chim**			0	0.381	0.743	0.742	0.88	0.913	0.922
**C.chim**				0	0.766	0.773	0.903	0.949	0.981
**F.whale**					0	0.347	0.868	0.906	1.012
**B.whale**						0	0.914	0.898	0.990
**Rat**							0	0.74	0.955
**Mouse**								0	0.859

**Fig 4:**
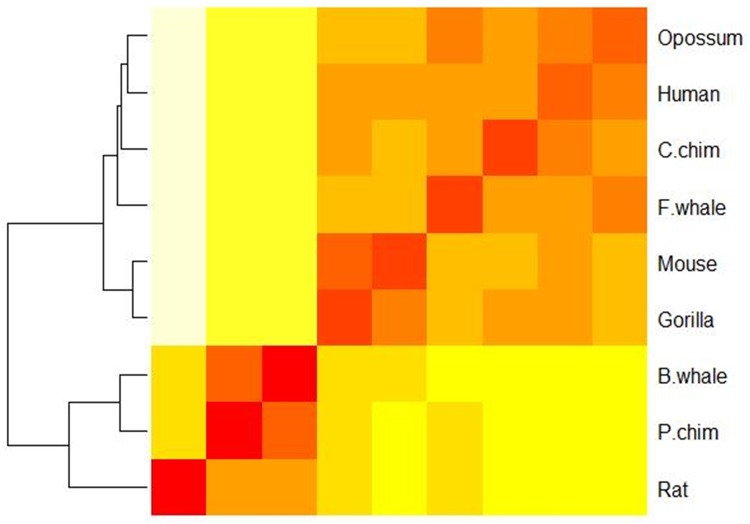
A heat map showing the similarity of nine species in the ND5 dataset based on the 20-D amino acid position ratio vector.

**Fig 5:**
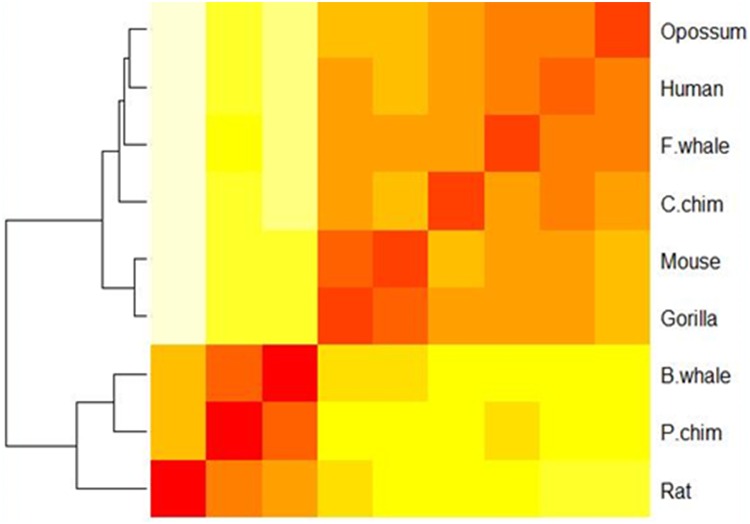
A heat map showing the similarity of nine species in the ND5 dataset based on the 20-D amino acid content ratio vector.

**Fig 6:**
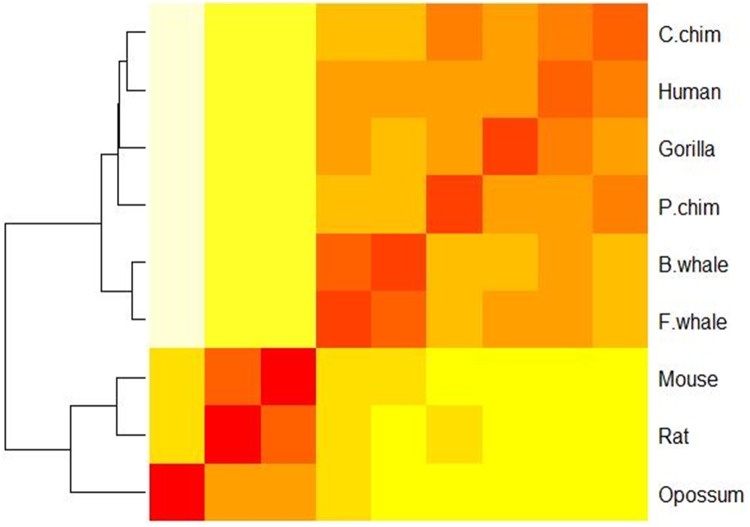
A heat map showing the similarity of nine species in the ND5 dataset based on the 40-D amino acid position ratio and content ratio vector.

In order to estimate the contribution of each part in the 440-D feature vector to the final performance in sequence similarity analysis, we plotted heat maps for the ND5 dataset based on the 20-D amino acid position ratio vector (see Fig 4), the 20-D amino acid content ratio vector (see Fig 5), and the 40-D amino acid position ratio and content ratio vector (see Fig 6), respectively. Clearly, Fig 3 is most consistent with the known result from the 440-D vector, Fig 6 is a little bit worse, and Figs 4 and 5 are the worst. As an indication, the 400-D Pseudo-Markov transition probability vector plays major role in sequence comparison.

A common strategy to evaluate an alignment-free method is to compare it with a popular alignment method like ClustalW [31], which has a much higher time and space complexity than alignment-free methods. Table 3 showed the pair-wise distances of the 9 protein sequences using ClustalW (i.e. Table 4 in [31]). We calculated the correlation coefficient between the distances from our method and those from ClustalW and compared our method with a few popular alignment-free methods [15, 31–34] using this coefficient as a criterion (see Table 4).

**Table 3:** The distance matrix of nine species calculated by ClustalW (i.e. Table 4 in [31]).

	Human	Gorilla	P.chim	C.chim	F.whale	B.whale	Rat	Mouse	Opossum
**Human**	0	10.7	7.1	6.9	41.0	41.3	50.2	48.9	50.4
**Gorilla**		0	9.7	9.9	42.7	42.4	51.4	49.9	54.0
**P.chim**			0	5.1	40.1	40.1	50.2	48.9	50.1
**C.chim**				0	40.4	40.4	50.8	49.6	51.4
**F.whale**					0	3.5	45.3	46.8	52.7
**B.whale**						0	45.0	45.9	52.7
**Rat**							0	25.9	54.0
**Mouse**								0	50.8

**Table 4:** Comparison of 6 alignment-free methods.

Method	Correlation coefficients
Our method	0.962
Ma et al. [31] (Table 3*)	0.9304
Matty et al. [32] (Table 3*)	0.6594
Bielińska-Wąż [34] (Table 4*)	0.7280
Wen et al. [33] (Table 3*)	0.7324
Yao et al. [15] (Table 3*)	0.6908

*The table in the literatures listed the correlation coefficient between the corresponding method and ClustalW.

As Table 4 shows, the correlation coefficient between our method and ClustalW is 0.962, which is the highest among the 6 methods. As a result, our method is more consistent with ClustalW than the other 5 methods, which indicates that our method is more accurate.

### Application to the F10 and G11 dataset

**Fig 7:**
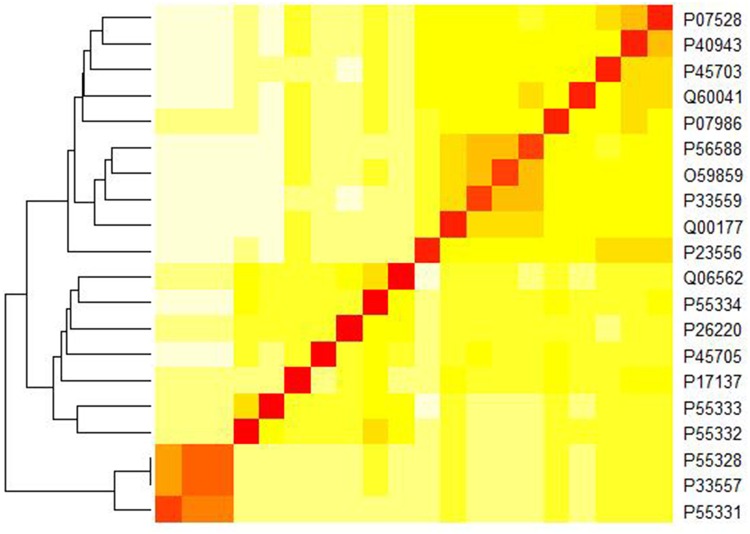
A heat map showing the similarity of 20 xylanases in the F10 and G11 datasets.

We also tested our method on the F10 and G11 datasets and plotted the heat map based on the pair-wise Euclidean distances in Fig 7. As can be seen, our method accurately separated the sequences in family F10 with those in G11 with the F10 xylanases locating in the top right quarter and G11 xylanases in the lower left quarter. We also observed that the F10 dataset is more heat stable than the G11 dataset, which is consistent with other studies, e.g.,[15].

It is of note that we applied the Euclidean distance in quantifying the distances among the feature vectors for different proteins. Euclidean distance is one of the simplest and most intuitive distance measures, which has been adopted in many fields, such as gene identification [35], protein 3D structure reconstruction [36], robust automatic pectoral muscle segmentation [37] and classification of normal and epileptic seizure EEG signals [38], etc. However, there are many other distance measures, which could affect protein similarity analysis. As an example, we compared the Euclidean distance with the Hamming distance for the ND5 dataset and F10 and G11 datasets respectively. We also plotted the heat-map for the ND5 dataset in S1 Fig based on the Hamming distance. Similar plots for the F10 and G11 datasets were shown in S2 Fig. Interestingly, Fig 3 and S1 Fig are almost the same while Fig 7 and S2 Fig exhibit significant differences. Clearly, Fig 7 (based on the Euclidean distance) is better since the two xylanases families are well separated while S2 Fig (based on the Hamming distance) fails to do it. For the ND5 dataset, we further computed the agreement (i.e., the Pearson correlation coefficients between the protein similarity matrices) between our method (based on the Hamming distance) and ClustalW, which is 0.937, a little bit less than that for the Euclidean distance (0.962). Thus, we believe that the Euclidean distance is more effective than Hamming distance for these two datasets.

## Conclusion

In this paper, we have proposed a novel alignment-free method to compare protein sequences. The method is more accurate than 5 other popular alignment-free methods in the ND5 dataset and is capable of distinguishing the F10 xylanases family from the G11 family. The comparison results of this method are quite consistent with protein sequence aligners like ClustalW. It presents an alternative of these aligners when time and space complexities become an issue.

In the future, a few machine learning methods [39] could be applied to further improve the performance of our method. For example, in contrast to phylogenetic analysis, methods like K-means analysis [40] and random forest [41] could also be applied to classify the proteins and perform taxonomy. However, it is out of the scope of this study. In addition, our novel features could also be applied into applications like essential gene identification [42] and similar problems related to DNAs or RNAs.

## Supporting Information

S1 Fig A heat map showing the similarity of nine species in the ND5 dataset based on the Hamming distance. (TIF)

S2 Fig A heat map showing the similarity of 20 xylanases in the F10 and G11 datasets based on the Hamming distance. (TIF)

S1 Table The nine ND5 protein sequences. (TXT)

S2 Table The 10 sequences in the F10 xylanase family. (TXT)

S3 Table The 10 sequences in the G11 xylanase family. (TXT)
