# Supplementary figures and images for: An Alignment-Free Algorithm in Comparing the Similarity of Protein Sequences Based on Pseudo-Markov Transition Probabilities among Amino Acids
Source: PLoS One. 2016 Dec 5;11(12):e0167430. doi: 10.1371/journal.pone.0167430 (PMC5137889; doi:10.1371/journal.pone.0167430)

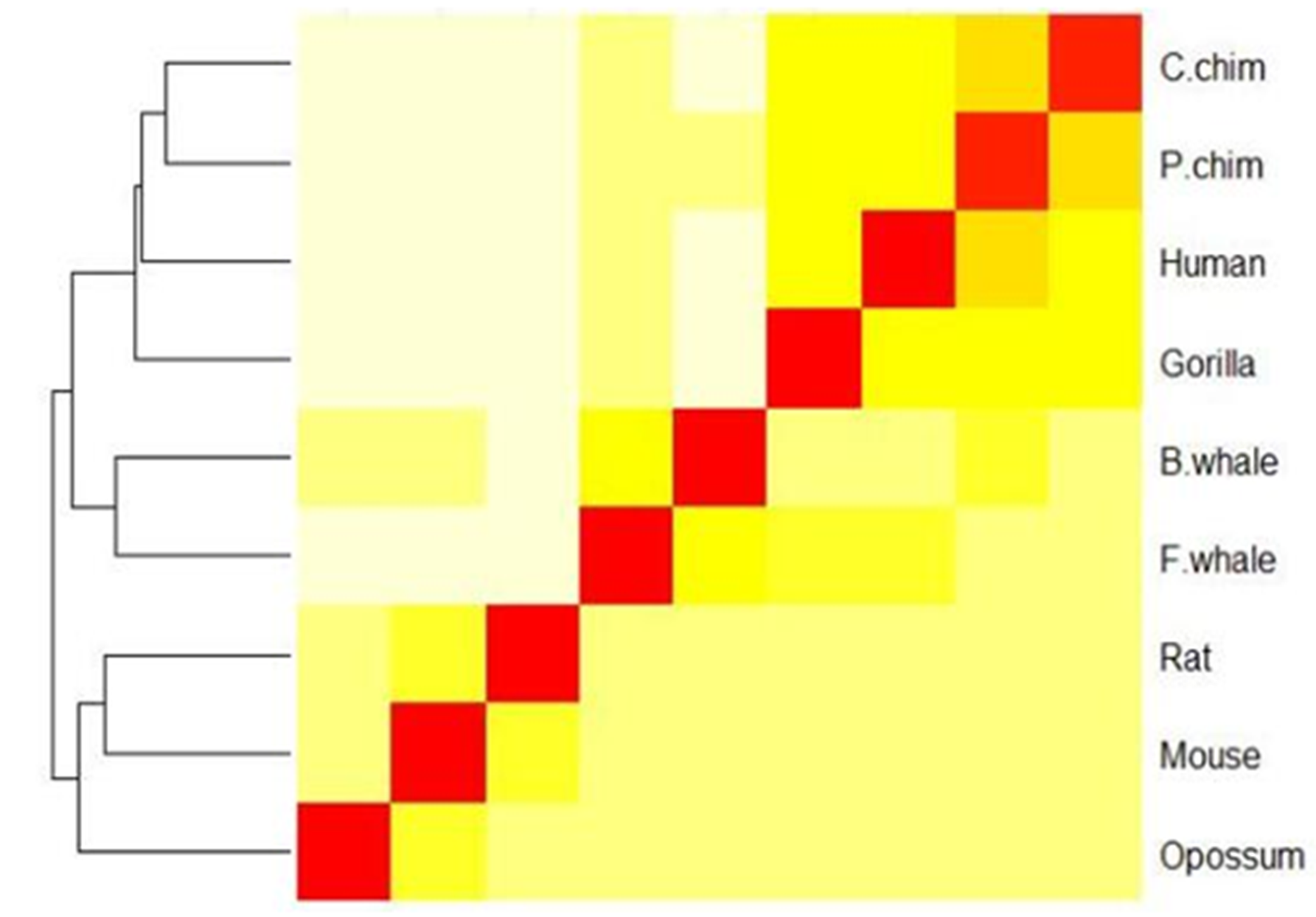

Supplement: S1 Fig — (TIF) [file pone.0167430.s001.tif]

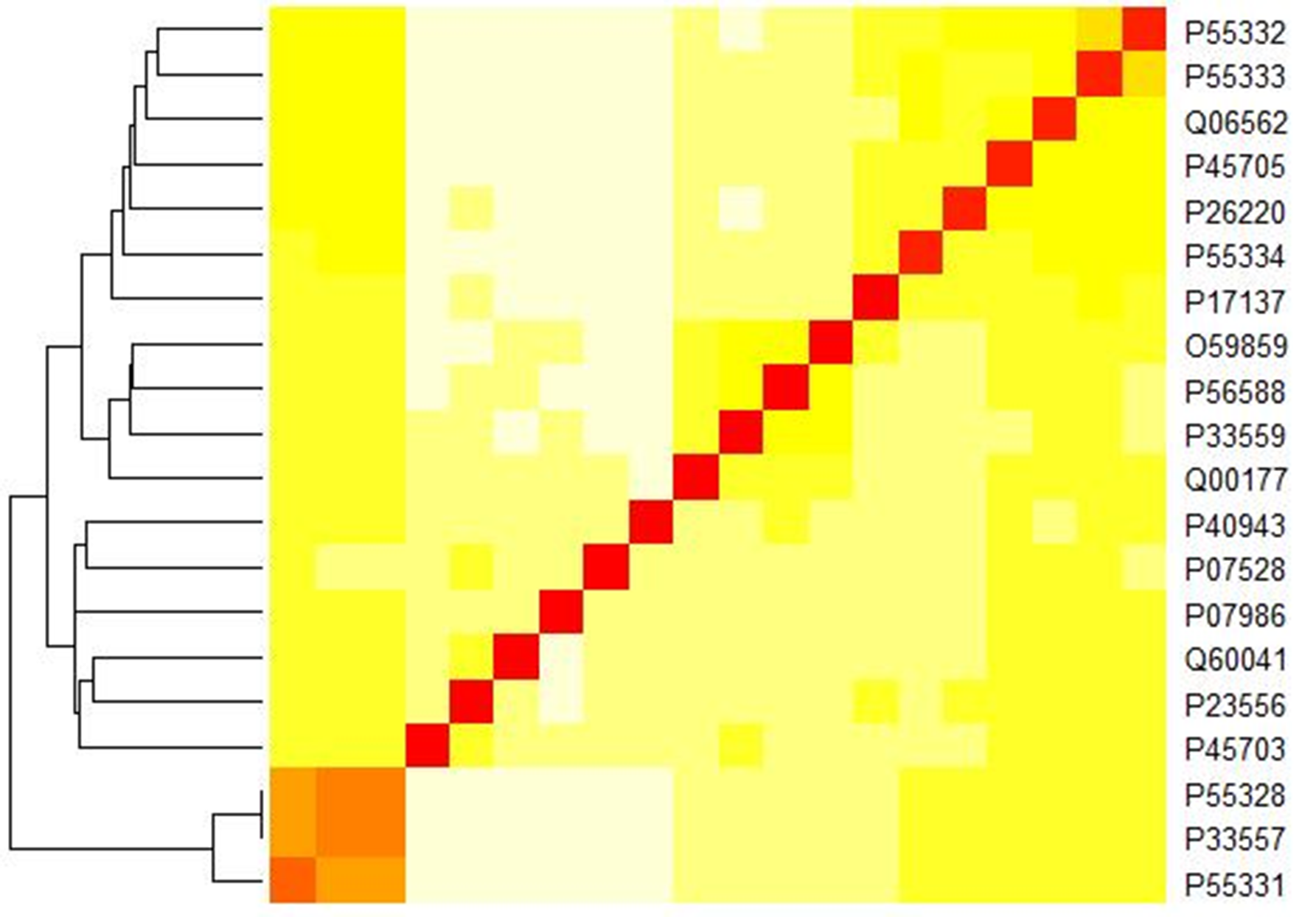

Supplement: S2 Fig — (TIF) [file pone.0167430.s002.tif]
